# Supplementary material for: Risk factors associated with Hepatitis B virus infection among pregnant women attending public hospitals in Addis Ababa, Ethiopia
Source: PLoS One. 2023 Apr 26;18(4):e0284646. doi: 10.1371/journal.pone.0284646 (PMC10132657; doi:10.1371/journal.pone.0284646)
Supplement: Attachment — Submitted filename: Comments and sugestion for review.docx [file pone.0284646.s001.docx]

The research done by Tesfu et al. on risk factors associated with Hepatitis B virus infection among pregnant women has public health importance and screened large sample size about 12,138 pregnant women. So it good and in the range of PLOS One journal. However there are points must be corrected before publication.

1. RISk factor is written in capital letters, please correct it Risk factor.
2. Why do you exclude HIV infected patents, because sexual transmitted infection as one of risk factor in your study?
3. In line 125-126, correct in this way. Variables significant at p <0.25 with the dependent variable in **bivariate analysis** were selected for multivariable analysis.
4. In result part, in table one marital status and religion, it says others, you should have to specify below in the table as foot note.
5. I have seen your recommendation but you have not specified the stake, please try to make that.

Major comment

1. In the method part, sample size part, there is no sample size calculation. How do you reach 300 case and 300 control , it is not clear?
2. What is your sampling technique; it is not indicated in the method part? Is it quota sampling or random what?
3. You have used predesigned and pre-tested, where do you pre-test your questionnaire, it is not mentioned well?
4. The test used for the diagnosis of HBsAg is rapid test for the selection of cases and controls which is weak for this purpose , it would have been good to use ELISA or PCR?
