# Supplementary material for: Risk factors associated with Hepatitis B virus infection among pregnant women attending public hospitals in Addis Ababa, Ethiopia
Source: PLoS One. 2023 Apr 26;18(4):e0284646. doi: 10.1371/journal.pone.0284646 (PMC10132657; doi:10.1371/journal.pone.0284646)
Supplement: Attachment — Submitted filename: Response to reviewers.docx [file pone.0284646.s002.docx]

**Rebuttal letter**

**Manuscript Number:** PONE-D-22-22777

**Manuscript title**: Risk factors associated with Hepatitis B virus infection among pregnant women attending public hospitals in Addis Ababa, Ethiopia

**Response to reviewers**

**Dear Dr. Yury E Khudyakov ( Acadamic editor of PLOS ONE journal)**

Thank you for giving us the opportunity to submit a revised manuscript of**^<<^** Risk factors associated with Hepatitis B virus infection among pregnant women attending public hospitals in Addis Ababa, Ethiopia ^>>^for publication in the journal of PLOS ONE. We appreciate the time and effort that you and the reviewers dedicated to provide feedback on our manuscript and are grateful for the insightful comments on and valuable improvements to our paper .We have incorporated almost all of the suggestions made by the reviewers. Those changes are marked within the manuscript. Please see below, in blue, for a point-by-point response to the reviewers’ comments.

**Academic editors and Reviewer’s comment to the authors**

1. RISk factor is written in capital letters, please correct it Risk factor.

**Author response: We agree and corrected it.**

2. Why do you exclude HIV infected patents, because sexual transmitted infection as one of risk factor in your study?

**Author response: Since HIV and HBV have similar route of transmission the risk factors may be over lapped that is why we exclude HIV seropositive pregnant woman.**

3. In line 125-126, correct in this way. Variables significant at p <0.25 with the dependent variable in bivariate analysis were selected for multivariable analysis.

**Author response: we accepted the reviewers comment and corrected it.**

4. In result part, in table one marital status and religion, it says others, you should have to specify below in the table as foot note.

**Author response: we agree and corrected it.**

5. I have seen your recommendation but you have not specified the stake, please try to make that.

**Author response: we agree and revised it.**

**Major comment**

1. In the method part, samplesize part, there is no sample size calculation. How do you reach 300 case and 300 control, it is not clear?

Author response: we accept the comment and corrected it as follow

To determine the MTCT of HBV and the protective effect of the immune-prophylaxis vaccine in Ethiopia, a sample size of 369 HBV-positive pregnant women was calculated using, 40% of MTCT of HBV in the absence of post-exposure prophylaxis in Nigeria, and giving any particular outcome to be with 5% marginal error, 95% confidence interval, and using the following single population determination formula.

n = (z ∂/2) ^2^ p (1-p)

d^2^

## To get 369 HBsAg positive, 12,318 pregnant women were screened for HBsAg as routine antenatal care (ANC) service in the selected public hospitals during the study period, and 300 of them were included in this study. The women whose screening results were positive for HBsAg were considered as cases. The purposive sampling technique was used to get the cases, and, for each HBsAg positive woman, the next HBsAg negative woman was interviewed and acted as a control. Thus 300 cases and 300 controls were involved in the study.

2. What is your sampling technique; it is not indicated in the method part? Is it quota sampling or random what?

**Author response**: **The purposive sampling technique was used to get the cases, and, for each HBsAg positive woman, the next HBsAg negative woman was interviewed and acted as a control.**

3. You have used predesigned and pre-tested, where do you pre-test your questionnaire, it is not mentioned well?

**Author response:** we accept the comment and revised it as follow

Questionnaires were carefully designed and pre-tested with individuals equivalent to 5% of the calculated sample size in Ras Desta Damtew Hospital and were slightly amended after pre-tested results revealed a lack of clarity for some responses.

4. The test used for the diagnosis of HBsAg is rapid test for the selection of cases and controls which is weak for this purpose; it would have been good to use ELISA or PCR?

**Author response: we agree also with this comment .But we could not do those tests because they were not easily accessible and we describe this as a limitation of the study.**

Once more we thank you for the comments. We believe our manuscript is now ready for publishing.

Sincerely,

Mebrihit Arefaine

On behalf of all authors
