# Supplementary material for: Risk factors associated with Hepatitis B virus infection among pregnant women attending public hospitals in Addis Ababa, Ethiopia
Source: PLoS One. 2023 Apr 26;18(4):e0284646. doi: 10.1371/journal.pone.0284646 (PMC10132657; doi:10.1371/journal.pone.0284646)
Supplement: Attachment — Submitted filename: Response to reviewers.docx [file pone.0284646.s003.docx]

**Rebuttal letter**

**Manuscript Number:** PONE-D-22-22777R1

**Manuscript title**: Risk factors associated with Hepatitis B virus infection among pregnant women attending public hospitals in Addis Ababa, Ethiopia

**Response to reviewers**

**Dear Dr. Yury E Khudyakov ( Acadamic editor of PLOS ONE journal)**

Thank you for giving us the opportunity to submit a revised manuscript of**^<<^** Risk factors associated with Hepatitis B virus infection among pregnant women attending public hospitals in Addis Ababa, Ethiopia ^>>^for publication in the journal of PLOS ONE. We appreciate the time and effort that you and the reviewers dedicated to provide feedback on our manuscript and are grateful for the insightful comments on and valuable improvements to our paper .We have incorporated all of the suggestions made by the reviewers. Those changes are marked within the manuscript. Please see below, in blue, for a point-by-point response to the reviewers’ comments.

**Academic editors and Reviewer’s comment to the authors**

1. Risk factors associated with HBV status among pregnant women are common for acquisition of HBV infections. The discussion section would be improved by comparing to risk factors in general population.

**Authors’ response: We agree and we included and comparing the risk factors of HBV infection of the pregnant women with the general population.**

2. This is a research paper rather than policy and cannot contain recommendations.  The observations made in the study, however, may be used to indicate importance of the identified factors for enhancing screening for HBV infections among pregnant women. Since the study did not involve application of vaccines and immunoglobulins, it is not proper to conclude on their administration.

**Authors’ response: we agree and corrected it.**

3. It is not sufficient to mention the use of low sensitivity assays in Limitations. Potential effects of these assays on the presented findings should be spelled out.

**Authors’ response: we agree and corrected as ^<<^** Due to a lack of laboratory setups, the HBsAg status of the pregnant women was not confirmed using an enzyme-linked immunosorbent assay, and there might be false positive or false negative results. In addition, since the polymerase chain reaction test was not carried out in this study, pregnant women with occult HBV infections were not identified.^>^.

Once more we thank you for the comments. We believe our manuscript is now ready for publishing.

Sincerely,

Mebrihit Arefaine

On behalf of all authors.
